# Predicting Treatment of Bioprosthetic Aortic Valve Failure in the United States: A Proposed Model

**DOI:** 10.1016/j.shj.2024.100339

**Published:** 2024-07-10

**Authors:** Philippe Généreux, Martin B. Leon, Roy D. Dar, Rishi Puri, Yoseph Rozenman, Molly Szerlip, Pradeep K. Yadav, Vinod H. Thourani, Philippe Pibarot, Danny Dvir

**Affiliations:** a Gagnon Cardiovascular Institute, Morristown Medical Center, Morristown, New Jersey; b Cardiovascular Research Foundation, New York, New York; c NewYork-Presbyterian Hospital/Columbia University Irving Medical Center, New York, New York; d Pi-Cardia, Rehovot, Israel; e Cleveland Clinic, Cleveland, Ohio; f Adelson School of Medicine, Ariel University, Ariel, Israel; g Baylor Scott & White The Heart Hospital Plano, Plano, Texas; h Marcus Valve Center, Piedmont Heart Institute, Atlanta, Georgia; i Quebec Heart and Lung Institute, Quebec City, Canada; j Department of Cardiology, Shaare Zedek Medical Center and Faculty of Medicine, Hebrew University of Jerusalem, Jerusalem, Israel

## Abstract

• Transcatheter aortic valve replacement (TAVR) is the dominant treatment for aortic stenosis; the need to treat TAVR failure is increasing.
• TAVR valve-in-valve (ViV) is predicted to reach ∼42,000 procedures by 2035.
• TAVR ViV will represent ∼15% of all TAVR performed by 2035.
• TAVR-in-TAVR will match TAVR-in-surgical aortic valve replacement by 2028 and dominate thereafter.
• To ensure the viability of TAVR ViV, the use of leaflet modification is needed.

Transcatheter aortic valve replacement (TAVR) and surgical aortic valve replacement (SAVR) are two recognized therapies for aortic stenosis (AS). Similarly, the use of a transcatheter aortic valve inside a failed surgical or transcatheter aortic valve has become an established strategy to treat patients. These valve-in-valve (ViV) procedures can be challenging and carry some inherent procedural risks, especially with regard to coronary obstruction, future coronary access, and valve performance. Understanding the trends in TAVR and SAVR utilization is important to predict the use of ViV TAVR in the future, which are major determinants for the lifetime management of AS. The aim of this study is to describe the recent rate of TAVR and SAVR use and, using current available data, to present a model to predict the use of transcatheter ViV procedures in the future.

## Methods

Using data from the Society of Thoracic Surgeons/American College of Cardiology Transcatheter Valve Therapies (TVT) Registry along with literature sources, we developed a Monte Carlo-based forecast model (The Math Works, Inc, MATLAB, version R2021b). This model incorporates inputs for annual TAVR and biological SAVR use and aims to forecast the annual volume of ViV TAVR procedures in the United States up to 2035. The following variables were included in our model:

1) **TAVR and SAVR.** Annual TAVR volume from 2012 to 2022 was sourced from the TVT registry and linearly extrapolated back to 2007 and forward to 2035, assuming an unsaturated market. Annual bioprosthetic SAVR volume relied on prior publications for the years 1998-2011 and leveraged the total aortic valve surgery volume from the Society of Thoracic Surgeons registry data for 2012-2020, assuming 80% bioprosthetic valve usage.^1^, ^2^, ^3^ Annual bioprosthetic SAVR volume was assumed to be constant at 50,000 cases from 2021 to 2035.
2) **Age.** The model populates procedures with patient ages ranging from 60 to 95 years, following a known age distribution, both for the initial TAVR and SAVR.^4^ The age distribution in the model aligns with two literature sources, ensuring consistency before and after 2017.^4^^,^^5^
3) **Patient life expectancy.** Life expectancy for SAVR patients postprocedure was assumed to match 85% low-risk and 15% intermediate-to-high-risk patients.^6^ TAVR patient life expectancy matched 100% intermediate to high-risk for 2014 to 2019, 2/3 intermediate to high-risk and 1/3 low-risk from 2019 to 2024, and 50% intermediate to high-risk and 50% low-risk for 2024 to 2035 (from the TVT registry and expert consensus).
4) **Modeling valve durability and survival.** For each patient, the model randomly selected a valve degeneration year postindex procedure based on a durability distribution and estimated patient survival postprocedure using the patient age at the index procedure and reported survival curves. Patients whose valve durability exceeded their survival date were removed from the pool of potential ViV candidates in the model. TAVR durability was assumed to be bimodal, with 20% early failure (4, standard deviation = 1.5 years),^7^^,^^8^ and 80% late failure (11.5, standard deviation = 3.5 years). Bioprosthetic SAVR durability was considered bimodal and age dependent,^9^ with distributions of 10 years for age at time of SAVR of <70, standard deviation = 5, and 17 years for age ≥70, standard deviation = 5. Median survival of 6 years, standard deviation = 2, was used for high-intermediate risk, and the low-risk survival curve matched reported values.^6^
5) **Redo-Procedures.** Redo-procedures such as SAVR-after-SAVR or SAVR-after-TAVR were excluded from the pool of potential patients for TAVR-in-SAVR or TAVR-in-TAVR procedures, respectively; they were accounted for using a 2023 report and extrapolated trends.^10^
6) **Penetration.** Penetration was fit to achieve the best fit between the total ViV and the observed TVT data. Among patients with TAVR failure, TAVR-in-TAVR treatment penetration was 10% up to 2022, with a progressive increase up to 60% in 2035, and for patients with SAVR failure, the treatment penetration was assumed to be 60% until 2022, with a progressive increase up to 80% in 2035.
7) **Model input processing.** To forecast annual ViV TAVR procedures, ages of valve patients were allocated to all TAVR and biological SAVR cases that were inputted based on reported age distributions. Next, valve durability distributions and survival distributions postprocedure were sampled to determine the valve expiration relative to the survival of each patient in the model. Patients who died before their valve expired were removed from the model. Finally, penetration was applied to the returning patients, and redo surgical procedures were removed from the model before integrating total annual TAVR-in-TAVR and TAVR-in-SAVR for each year to 2035. Simulation models were run 20 times using the same input parameter set, with a calculated total ViV standard deviation of 110 cases for 2020 and 240 cases for 2035.

## Results

**Figure 1:**
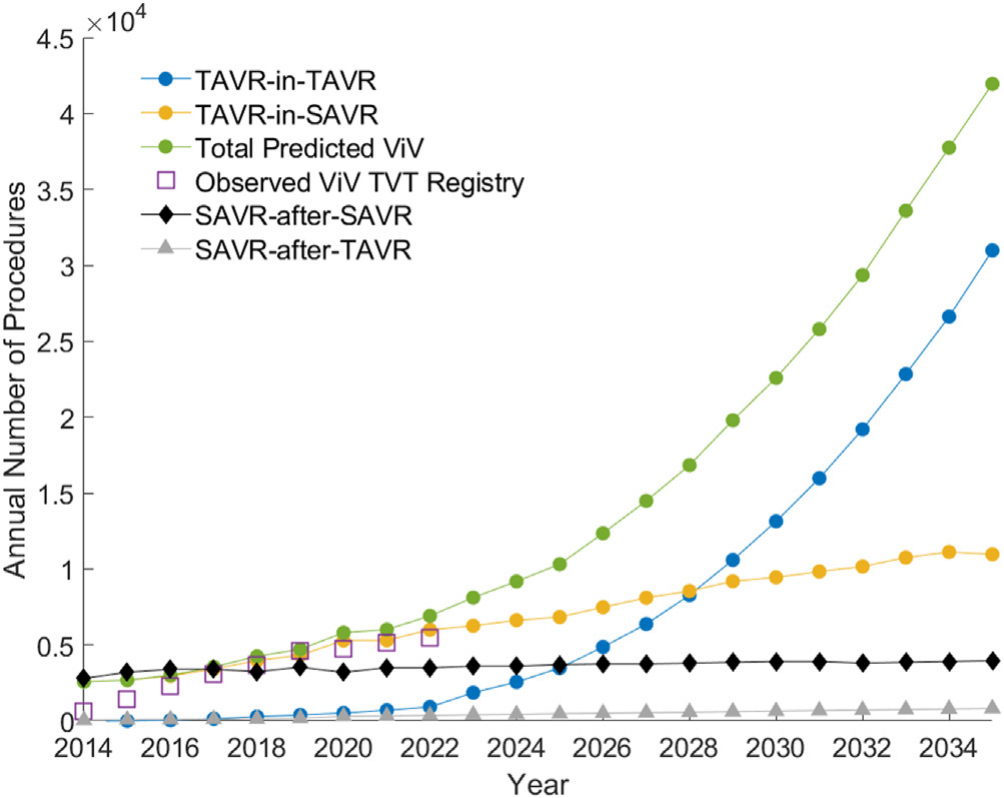
Predictive model valve-in-valve (ViV). Annual model forecast output from the simulation. Annual total ViV volume (green curve) matches observed TVT registry data (purple squares) and is composed of the summation of the TAVR-in-TAVR (blue curve) and TAVR-in-SAVR (yellow curve). Redo procedure trends for SAVR-after-SAVR (black diamonds) and SAVR-after-TAVR (gray triangles) are also plotted. For total ViV in 2035, standard deviation = 240 cases. Abbreviations: SAVR, surgical aortic valve replacement; TAVR, transcatheter aortic valve replacement; TVT, Transcatheter Valve Therapies.

The model generates outputs for total ViV procedures, including total TAVR-in-SAVR and TAVR-in-TAVR for each year (Figure 1). The goal was to fit the model’s predicted ViV volume to the known annual United States total ViV data from the 2023 TVT Registry. Our derived model for the United States shows strong alignment with the 2023 TVT Registry ViV data. Interestingly, we observed that TAVR-in-SAVR represents the most frequent type of ViV up until 2022, yet is expected to plateau thereafter. Conversely, TAVR-in-TAVR begins to rise slowly in 2023 and is expected to grow dramatically afterward. TAVR-in-TAVR is predicted to nearly match TAVR-in-SAVR in 2028, and the total ViV is projected to reach approximately 42,000 procedures in 2035 (∼15% of all TAVR).

## Discussion

Over the last 10 years, TAVR has become the dominant treatment for AS. With the expansion to younger population, patients are expected to outlive their bioprosthetic valve and require a second procedure to treat their TAVR failure. Expectedly, TAVR-in-TAVR ViV will become more frequent. Our proposed predictive model demonstrates the exponential growth of TAVR-in-TAVR ViV over the next 10 years, paralleling the prior growth of TAVR over the previous 10 years. Our model predicts that a significant proportion of all the TAVR performed in the future will be ViV TAVR. Although our model used rigorous and contemporary assumptions, variability and unpredictability of some assumptions, including improvement in valve durability, implantation, or surgical technique, may affect the output of our model. That said, the impact of such factors can be minor, and the development, dissemination, and global adoption of such technologies are not expected to occur within the next 10 years. Similarly, our model neither account for potential TAVR expansion in patients younger than 60 years old nor for patients requiring a third intervention (i.e., TAVR-in-TAVR-in-TAVR).

In summary, TAVR has emerged as the dominant procedure to treat AS, especially among patients older than 60 years of age with suitable anatomy. As patients presenting with failed TAVR are becoming more frequent, TAVR inside a failed transcatheter aortic bioprosthesis is expected to represent a significant proportion of all TAVR procedures performed in the future. These data reinforce the need for ongoing development of adjunctive techniques and dedicated technologies to optimize transcatheter aortic ViV procedures.^11^

## Funding

Funding for this paper was provided by Pi-Cardia.

## Disclosure Statement

P. Généreux is a consultant and advisor for Abbott Vascular and receives speaker fees from Abbott Vascular; is an Abiomed consultant and advisor for Abiomed and receives speaker fees from Abiomed; is a Boston Scientific consultant; is a Cardiovascular System Inc consultant; PI Eclipse Trial; is an Edwards Lifesciences consultant and advisor, receives speaker fees from Edwards Lifesciences, is a proctor of Edwards Lifesciences, and receives research grant, PI EARLY-TAVR trial, PI PROGRESS trial; is a GE Healthcare consultant; is an iRhythm Technologies consultant; is a Medtronic consultant, advisor, and receives speaker fees from it; is an OpSens consultant; is a Pi-Cardia consultant and has equity; is a Puzzle Medical consultant and has equity; is Saranas: equity and consultant; Shockwave: consultant and receives speaker fees; Siemens: consultant; a Soundbite Medical Inc consultant and has equity; is a Teleflex consultant; is a 4C Medical consultant; PI ALTA Valve Feasibility Study; is a consultant and advisor for egnite, Inc. M. B. Leon receives institutional clinical research grants from Abbott, Boston Scientific, Edwards Lifesciences, and Medtronic. R. D. Dar is an employee of Pi-Cardia. R. Puri is a consultant, speaker, and proctor for Medtronic and Abbott; is a consultant for Centerline Biomedical, Philips, Products & Features (PI TRICAV 1 and 2 trials), Shockwave Medical, VDyne, VahatiCor, Advanced Nanotherapies (PI ADVANCE DCB 1), NuevoSono, TherOx, GE Healthcare, Anteris, T45 Labs, Pi-Cardia, Protembis, Nyra Medical; and has equity interest in Centerline Biomedical, VahatiCor, and NuevoSono. Y. Rozenman is an employee of Pi-Cardia. P. K. Yadav is a consultant, receives speaker fees, and is a proctor for Edwards Lifesciences, Abbott Vascular, and Boston Scientific; on the advisory board and has equity in Opus Medical and Dasi Simulations; and the PI ALLIANCE VIV trial by Edwards Lifesciences. V. H. Thourani is a researcher or consultant for Abbott Vascular, Atricure, Artivion, Boston Scientific, Dasi Simulations, Edwards Lifesciences, Egnite, and Medtronic. He has equity in Dasi Simulations. P. Pibarot receives institutional funding from Edwards Lifesciences, Medtronic, Pi-Cardia, Cardiac Success, and Roche Diagnostics for echocardiography core laboratory analyses, blood biomarker analyses, and research studies in the field of interventional and pharmacologic treatment of valvular heart diseases, for which he received no personal compensation. D. Dvir is a consultant to Edwards Lifesciences, Medtronic, Abbott, and Pi-Cardia and has equity in Pi-Cardia. The other author had nothing to disclose.
